# Real-World Adherence to Asthma and COPD Medications in Belgium: A Nationwide Analysis of Determinants Using Dispensing Data and Mixed-Effects Modeling

**DOI:** 10.3390/healthcare14080982

**Published:** 2026-04-09

**Authors:** Amélie Rosière, Sebastian Riemann, Olfa Guaddoudi, Stéphanie Pochet, Guy Brusselle, Carine De Vriese

**Affiliations:** 1 Department of Pharmacotherapy and Pharmaceutics, Faculté de Pharmacie, Université libre de Bruxelles (ULB), 1050 Brussels, Belgium; stephanie.pochet@ulb.be (S.P.); carine.de.vriese@ulb.be (C.D.V.); 2 Department of Respiratory Medicine, Ghent University Hospital, 9000 Ghent, Belgium; sebastian.riemann@ugent.be (S.R.); guy.brusselle@uzgent.be (G.B.); 3 Department of Epidemiology, Erasmus Medical Center, 3015 Rotterdam, The Netherlands; 4 Independent Consultant, Principal Statistical Programmer, 3890 Gingelom, Belgium; consulting.sam.group@gmail.com; 5 Department of Respiratory Medicine, Erasmus Medical Center, 3015 Rotterdam, The Netherlands

**Keywords:** therapeutic adherence, real-world data, asthma, COPD, inhaled therapies, medication utilization, mixed-effects models, pharmacoepidemiology

## Abstract

**Highlights:**

**What are the main findings?**

- Real-world adherence to asthma and COPD medications in Belgium was critically low, with only 30.5% of patient–treatment exposures reaching good adherence, and extremely poor adherence was observed among children, adolescents, and users of ICS or LABA + ICS combinations.
- Mixed-effects modeling identified age, sex, and pharmacological class as strong and independent predictors of adherence, with markedly higher adherence for triple therapy and oral treatments compared to ICS.

**What are the implications of the main findings?**

- The identification of high-risk groups—particularly children, adolescents, and ICS users—provides clear targets for tailored patient-centered interventions aimed at improving long-term medication continuity.
- Results support guideline-concordant strategies such as regimen simplification, enhanced inhaler technique training, and digital adherence tools to reduce the substantial adherence gap in chronic respiratory diseases.

**Abstract:**

**Background/Objectives**: Therapeutic adherence to asthma and COPD medications remains worryingly low and varies widely across patient groups, underscoring persistent challenges in chronic respiratory care. The aim of this nationwide study is to quantify real-world adherence and to identify its demographic and clinical determinants using the Belgian health care claims database of the National Institute for Health and Disability Insurance (NIHDI). **Methods**: Adherence was assessed using the Continuous Multiple Interval Measure of Medication Availability (CMA) among patients treated between 2020 and 2023. Mixed-effects logistic regression was applied to identify determinants of adherence. **Results**: Only 30.5% of patients achieved good adherence (CMA ≥ 0.8). Adherence varied substantially across pharmacological classes, ranging from 8.1% for inhaled corticosteroids to 66.4% for triple therapy. Age emerged as a major determinant, with adherence increasing steadily across age groups: only 4.0% of children and 15.7% of adolescents reached good adherence, compared with progressively higher rates in adults. Mixed-effects logistic regression confirmed age, sex, and pharmacological class as robust predictors of adherence. **Conclusions**: These findings highlight the magnitude of the therapeutic adherence gap in chronic respiratory diseases and clearly identify children, adolescents, and ICS or LABA + ICS users as the highest-risk groups. Recognizing these profiles has direct implications for clinical practice, as it provides concrete targets for future patient-centered interventions and guideline-concordant adherence-enhancing strategies.

## 1. Introduction

Asthma and chronic obstructive pulmonary disease (COPD) are highly prevalent chronic respiratory diseases worldwide and represent a major burden for healthcare systems [1,2,3]. The clinical management of asthma and COPD is guided by international evidence-based recommendations, notably the Global Initiative for Asthma (GINA) [4] and the Global Initiative for Chronic Obstructive Lung Disease (GOLD) [5]. These guidelines emphasize the importance of appropriate pharmacological therapy to achieve disease control and prevent exacerbations. However, once optimal treatment is prescribed, a critical challenge remains: ensuring optimal therapeutic adherence, starting from treatment initiation and continuing throughout the disease course.

Poor adherence has been linked to reduced quality of life and greater symptom burden and disease progression, as well as more frequent exacerbations, hospitalizations, and increased healthcare costs [6,7,8,9,10,11]. Addressing this public health concern requires robust quantification of adherence and identification of its determinants [12,13].

Although therapeutic adherence has been widely studied, many previous analyses suffered from methodological heterogeneity and limited transparency in adherence quantification, leading to non-comparable and sometimes contradictory findings [14,15]. To overcome these limitations, the present study applies a standardized and reproducible measure of adherence—the Continuous Multiple-Interval Measure of Medication Availability (CMA)—which enables rigorous longitudinal tracking of medication use based on dispensing data [12,13]. Beyond this standardized quantification, a major strength of this work lies in its analytical framework: CMA was combined with robust multivariable mixed-effects logistic regression models, incorporating patient-level random effects to account for repeated observations across pharmacological classes. This approach reflects real-world treatment trajectories, as individual patients may switch, escalate, or de-escalate between medication classes depending on disease evolution or clinical reassessment. By modeling within-patient variability, the mixed-effects structure provides a more realistic and precise estimation of adherence determinants. This dual methodological strategy enhances comparability and improves the precision of estimates, allowing for a reliable identification of demographic and clinical determinants of therapeutic adherence. By identifying the most vulnerable patient groups, this approach may help inform future research and guide the development of targeted strategies aimed at supporting long-term medication continuity.

This study provides the first nationwide, multivariable analysis of adherence to asthma and COPD treatments in Belgium during the 2020–2023 period, using a large population-based cohort and mixed-effects modeling. Unlike previous Belgian work focused exclusively on inhaled therapies, this analysis includes both inhaled and oral treatments, thereby offering a comprehensive comparison across pharmacological classes. By quantifying strong and independent effects of age, sex, and treatment class—including extremely low adherence in younger patients and marked disparities between inhaled corticosteroids and triple therapy—this study generates new and clinically relevant insights into real-world adherence patterns.

## 2. Materials and Methods

### 2.1. Study Design and Data Source

This nationwide cohort study used pseudonymized dispensing data from the Belgian NIHDI database, which records all reimbursed community-pharmacy dispensations of asthma and COPD medications.

### 2.2. Study Participants

Patients were eligible if they received ≥2 dispensations of R03 medications between January 2020 and December 2023, reflecting chronic use. Pharmacological classes recommended by the GINA and GOLD guidelines for the chronic treatment of asthma and COPD were retained for analysis. These included inhaled medications: for COPD, Long-Acting Beta-Agonist (LABA), Long-Acting Muscarinic Antagonist (LAMA), and LABA + LAMA combinations; for asthma, Inhaled Corticosteroids (ICS); for asthma or COPD, LABA + ICS combinations and ICS + LAMA + LABA triple therapy; as well as oral medications, montelukast. Although not recommended in the GINA guidelines, theophylline was included in the study due to its indication as a maintenance treatment for asthma.

### 2.3. Extracted Variables

Extracted variables included patient sex, predefined age category, dispensation date, CNK code, DDDs, number of packages, and reimbursement details.

Age was provided by the INAMI in predefined 5-year intervals (e.g., 11–15, 16–20, 21–25, etc.). For analytical purposes, these intervals were grouped into broader age classes as follows: 0–10 years; 11–20 years; 21–40 years; 41–65 years; >65 years.

### 2.4. Data Cleaning

All variables were systematically reviewed to identify clearly missing, erroneous or implausible values. There were no missing values in the dataset.

The following values were excluded:

- Negative reimbursement or negative copayment, which may arise from pharmacy-level administrative adjustments (e.g., correction of a full-price advance when the patient later provides the reimbursable prescription). These values do not correspond to actual medication supply events and were excluded.
- Negative or zero package counts, as dispensations must include at least one physical package; such values typically reflect prescription regularizations at the pharmacy or the purchase of the medicine by the pharmacist.
- More than 10 packages dispensed on the same date for the same medication, a threshold identified through exploratory data checks to capture outliers inconsistent with routine pharmacy practice.

Implausible values were identified through systematic variable-wise inspection and removed prior to calculating adherence metrics.

### 2.5. Adherence Measurement

To minimize edge effects, adherence was assessed within an Observation Window (OW) starting 45 days after the first dispensation and spanning 1370 days.

#### Medication Adherence Metric

Adherence was assessed using the CMA, computed with the AdhereR package (version 0.8.1). The specific metric used was CMA3. CMA3 is defined as:

$$CMA3\;=\frac{Sum\;of\;the\;durations\;of\;medication\;events,\;excluding\;the\;last}{Number\;of\;days\;between\;the\;first\;and\;last\;event}$$

CMA3 values were capped at 1 [12]. The duration corresponds to the number of days covered by the dispensed medication.

CMA3 was selected because it avoids overestimation of adherence by excluding the coverage provided by the last dispensing event. Since the observation period ends on the day of the last refill, including the days theoretically covered after that date would artificially inflate adherence. By not counting future coverage and by capping values at 1, CMA3 prevents bias from stockpiling and provides an appropriate conservative estimate for our 4-year OW.

CMA3 was computed for each patient–class pharmacological pair using AdhereR (v0.8.1), with duration derived from WHO-defined daily doses (DDDs). CMA3 values of ≥0.8 defined adherence.

Only patients with ≥3 dispensations within the OW contributed CMA estimates, because at least two refill intervals are required to calculate CMA3 once the OW has started 45 days after the first dispensing. For patients with fewer than three dispensations, CMA3 could not be computed and therefore appeared as missing. These missing CMA3 values were removed from the dataset used for the descriptive adherence analyses. No imputation was applied.

### 2.6. Descriptive Analysis

Descriptive statistics were computed to summarize the distribution of the CMA, including mean, standard deviation, median, and interquartile range (IQR). The CMA was dichotomized using the threshold of 0.80, and adherence proportions were calculated overall and stratified by age category, sex, pharmacological class, and administration route. Group differences were assessed using χ^2^ tests.

### 2.7. Statistical Model Specification

To estimate adjusted associations between patient characteristics and adherence, we fitted a multivariable mixed-effects logistic regression model. The dependent variable was adherence (CMA ≥ 0.80). Because patients can appear multiple times when exposed to multiple pharmacological classes, a random intercept for patient ID was included to account for within-patient correlation. The random-effects structure therefore consisted of a patient-level random intercept only (1|patient ID).

The fixed-effect structure included age category and sex based on clinical relevance and previous literature identifying these variables as key determinants of adherence [16]. Route of administration was excluded due to structural collinearity with pharmacological class (e.g., montelukast is exclusively oral; LAMA therapies are exclusively inhaled).

Model diagnostics were performed to assess the adequacy of the mixed-effects logistic regression. Given the large dataset, diagnostics for the random intercept were based on a random subsample of 5000 patient-level estimates to ensure computational feasibility. The distribution of these random intercepts was approximately symmetric, with no evidence of multimodality or extreme deviations. Multicollinearity among fixed effects was evaluated using generalized variance inflation factors (GVIF), all of which were below 1.05, indicating an absence of problematic collinearity. Overdispersion was assessed using the Pearson residual dispersion ratio (value: 0.83), which did not indicate substantial overdispersion. Overall, these diagnostics supported the adequacy of the model specification. Model significance was evaluated using Type III ANOVA (χ^2^ distribution).

All analyses were conducted using RStudio (R version 4.4.1) using data.table for data manipulation and the lme4 package for mixed-effects modeling.

#### Reference Categories

The following reference categories were selected for interpretability and clinical relevance:

- Age category: 21–40 years, representing a stable young-adult baseline against which adherence patterns in children, adolescents and older adults can be meaningfully compared.
- Sex: The male category was selected as the reference because women are consistently reported in the literature as being at higher risk of poor medication adherence [17,18]. Using males as the baseline therefore allows a more direct and intuitive interpretation of adherence differences among females, a key group of interest in this study.
- Pharmacological class: ICS, reflecting their central role in asthma management and their wide use across age strata [4].

### 2.8. Sensitivity Analysis

Sensitivity analyses recalculated adherence separately for each calendar year (2020–2023) to examine temporal stability and to reduce the risk of including acute, non-chronic treatment episodes. This yearly restriction increases specificity for chronic use and allows verification that observed adherence patterns do not depend on the initial FUW.

### 2.9. Ethical and Data Protection

Data access, pseudonymization, and oversight procedures were approved by the NIHDI Request Evaluation and by the Information Security Committee of the National Institute for Health and Disability Insurance (CSI–INAMI), with Data Protection Impact Assessment (DPIA) validation by the Data Protection Officer of the Université libre de Bruxelles (ULB).

## 3. Results

### 3.1. Baseline Characteristics

The initial cohort included all patients meeting eligibility criteria after data cleaning (*n* = 1,384,363). Baseline characteristics for this cohort are presented in Table 1.

The initial cohort was predominantly composed of adults aged 41–65 years (32.5%) and older adults over 65 (27.9%). The sex distribution was balanced, with 52.5% female patients.

Most patients received only inhaled medications (88.9%), while 2.9% were treated exclusively with oral drugs. A combined use of both oral and inhaled therapies during the Follow-Up Window (FUW) was observed in 8.1% of patients.

79.9% of patients were exposed to a single pharmacological class. An additional 15.4% received two distinct classes, while 4.8% were exposed to three or more.

Among all dispensations (*n* = 10,942,658) recorded in the initial cohort, LABA + ICS dual therapies represented the most frequently dispensed pharmacological class, accounting for nearly half of all dispensations (48.7%). ICS monotherapy accounted for 19.2% of dispensations. Among oral treatments, montelukast was the most dispensed, representing 11.8% of all dispensations. Triple therapies (ICS + LAMA + LABA) accounted for 6.3% of dispensations between 2020 and 2023. The remaining pharmacological classes each represented less than 5% of total dispensations: LABA (2.5%), LABA + LAMA (4.4%), LAMA (5%), and theophylline (2.1%).

For adherence assessment, Continuous Multiple-Interval Measure of Medication Availability 3 (CMA3) was computed within an OW starting 45 days after the first dispensation. Patients with fewer than three dispensations during OW could not have CMA3 calculated (see the Materials and Methods Section), resulting in missing values. The final study population therefore comprised 878,342 patients. Characteristics for this subset are presented in Table 2 (column “Total Exposures”).

### 3.2. Descriptive Adherence Outcomes

The mean CMA3 was 0.52 ± 0.35, with a median of 0.48 [IQR: 0.19–0.89], based on 1,103,386 CMA values. Adherence was defined as CMA ≥ 0.8; patients with CMA < 0.8 were considered non-adherent. Overall, 30.5% of patients were classified as adherent (CMA ≥ 0.8). Therapeutic adherence was evaluated across patient and treatment characteristics, including age, sex, route of administration, and pharmacological class (Table 2).

Age categories were defined as 0–10 years, 11–20 years, 21–40 years, 41–65 years, and >65 years. Adherence varied substantially by age, with the lowest rates among children (0–10 years; 4.0%) and adolescents (11–20 years; 15.7%). Young adults (21–40 years) showed 21.0% adherence, rising to 34.4% in middle-aged adults (41–65 years). Older adults (>65 years) had the highest adherence (43.7%), although rates remained below 50%.

Sex differences were minimal, with slightly lower adherence among females (29.9%) compared to males (31.1%). By administration route, adherence was markedly lower for inhaled therapies (27.1%) than for oral treatments (58.9%). Across pharmacological classes, the lowest adherence was observed for ICS (8.1%) and LABA + ICS combinations (23.5%), while LAMA (61.7%) and triple therapy ICS + LAMA + LABA (66.4%) showed the highest adherence rates. Oral treatments such as montelukast and theophylline had intermediate adherence (59.2% and 55.4%, respectively).

### 3.3. Findings from the Statistical Analysis

#### 3.3.1. Univariate Analysis

All tested variables (Sex, Age Category, Administration Route, and Pharmacological Class) showed statistically significant associations with adherence status (chi-square test, *p* < 0.001), indicating that demographic and treatment-related factors are likely to influence therapeutic adherence.

#### 3.3.2. Multivariable Analysis

The model revealed several statistically significant associations:

- Age Category: Adherence increased progressively with age. The youngest age groups showed lower odds of adherence (0–10 years: OR = 0.25, CI [0.24–0.26]; 11–20 years: OR = 0.59, CI [0.58–0.61]) compared with young adults (21–40 years). In contrast, middle-aged adults (41–65 years) (OR = 1.70, CI [1.67–1.73]) and older adults (≥65 years) (OR = 2.42, CI [2.38–2.46]) demonstrated significantly higher odds of adherence.
- Sex: Female patients had slightly lower odds of adherence than males (OR = 0.89, CI [0.89–0.90]).
- Pharmacological Class: Using ICS as the reference, all other classes were associated with higher adherence. The highest odds were observed for montelukast (OR = 11.02, CI [10.79–11.25]), triple therapy ICS + LAMA + LABA (OR = 9.09, CI [8.89–9.30]), and LAMA (OR = 7.08, CI 95% [6.90–7.25]).

**Figure 1:**
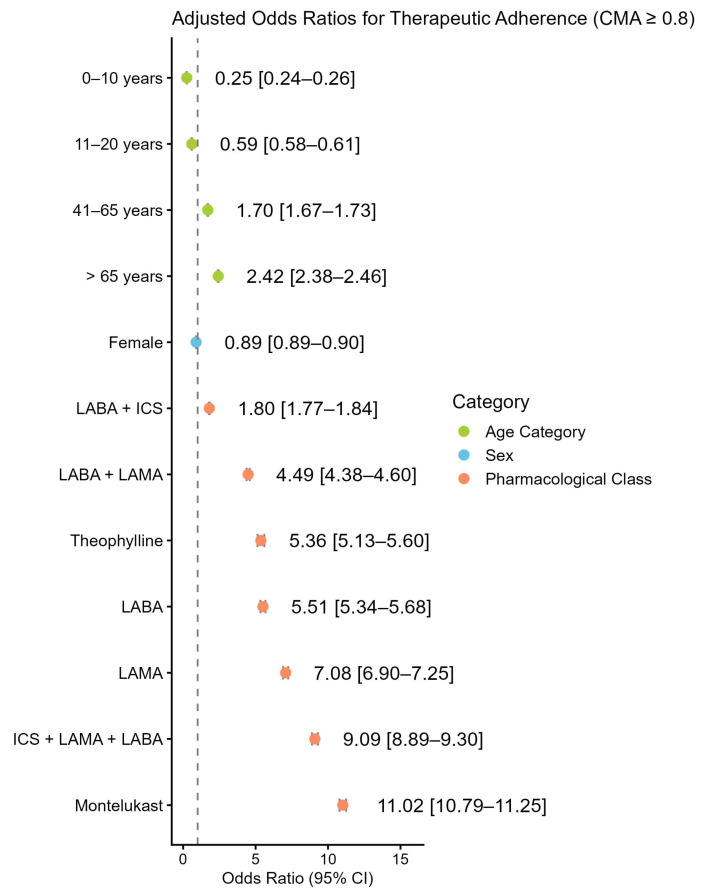
Forest plot showing adjusted odds ratios (OR) with 95% confidence intervals (CI) for variables (Age Category, Sex, Pharmacological Class) associated with therapeutic adherence (CMA ≥ 0.8). OR > 1 indicates increased odds of adherence. Reference categories: Age Category: 21–40 years; Sex: Male; Pharmacological Class: ICS. All associations presented are statistically significant (*p* < 0.001). Abbreviations: ICS: Inhaled Corticosteroids, LABA: Long-Acting Beta-Agonist, LAMA: Long-Acting Muscarinic Antagonist.

These results are visually represented by a forest plot in Figure 1 and are summarized in Appendix B Table A9.

- Type III ANOVA

To assess the overall contribution of each variable to the prediction of therapeutic adherence, a Type III ANOVA based on the chi-squared distribution was performed.

All tested variables—age category, sex, and pharmacological class—showed highly significant global effects (*p* < 0.001), indicating that each variable contributes meaningfully to the prediction of adherence.

This reinforces the robustness of the multivariate findings, especially given the large sample size and the narrow confidence intervals observed for the odds ratios. These results support the identification of these variables as key determinants of therapeutic adherence.

### 3.4. Sensitivity Analysis

Sensitivity analyses confirmed that adjusted odds ratios for age, sex, and pharmacological class remained stable when adherence was calculated within fixed 12-month windows instead of the full 4-year FUW. The direction and magnitude of associations were consistent across all calendar years, supporting the robustness of these determinants.

The higher adherence observed in the annual sensitivity analyses is anticipated and reflects the shorter FUW (Appendix A Table A1, Table A2, Table A3, Table A4, Table A5, Table A6, Table A7 and Table A8). Applying the same inclusion criterion (≥3 dispensations) within a 12-month FUW inherently selects patients with more regular refill behavior, making it easier to achieve CMA ≥ 0.8. In contrast, the 4-year FUW captures long-term treatment interruptions, gaps and discontinuation, which mechanically lowers adherence estimates.

## 4. Discussion

In this nationwide cohort of more than 870,000 patients, therapeutic adherence to asthma and COPD maintenance treatments was critically low, with only 30.5% achieving CMA ≥ 0.8. This aligns closely with concerns raised in both GINA and GOLD, which highlight poor adherence as a major barrier to effective long-term respiratory disease control. Prior studies similarly report the clinical consequences of non-adherence, including poorer symptom control, reduced quality of life, and increased healthcare and societal costs driven by more frequent exacerbations and hospitalizations [6,7,8,10,11,16,19,20].

Beyond confirming previously described patterns, this study provides the first nationwide, multi-year assessment of adherence to asthma and COPD medications in Belgium using standardized CMA3 and mixed-effects modeling. The granularity of the data allows identification of high-risk subgroups (children, adolescents, and ICS users) and comparative adherence across pharmacological classes. These adherence estimates are based on pharmacy dispensing records rather than confirmed medication intake. Accordingly, the patterns described reflect dispensing behavior, and the observed trends should be interpreted within this context.

Age emerged as the strongest demographic determinant of adherence. Children and adolescents demonstrated extremely low adherence (4.0% and 15.7%), consistent with earlier studies reporting limited autonomy, parental concerns, and challenges related to inhaler technique [21,22,23]. These findings strongly echo GINA, which emphasizes that adolescents exhibit some of the poorest adherence in asthma due to stigma, intentional non-adherence, and perceived lack of benefit. These early life patterns carry significant clinical implications, as pediatric non-adherence is associated with poor asthma control and reduced lung function [11,24]. Adolescents face additional barriers, including organizational difficulties and low perceived treatment benefit [25,26]. Recent evidence highlights the complex interplay between clinical, organizational and demographic determinants in pediatric populations, leading to higher care needs, increased coordination requirements and greater vulnerability to suboptimal outcomes [27]. These findings parallel our observation of very low adherence among children and adolescents, suggesting that the broader care complexity associated with pediatric chronic conditions may contribute to the adherence gap in this age group.

In contrast, adherence increased progressively with age and peaked among adults older than 65 years, although it remained below 50%. This pattern is consistent with GOLD, which notes that COPD—predominantly affecting individuals older than 40 years—is characterized by persistent daily symptoms that may encourage more regular medication use. Conversely, asthma, particularly allergic or intermittent phenotypes common in younger patients, presents with fluctuating symptom burden, leading some patients to perceive less need for consistent controller therapy [28]. Nonetheless, age-related barriers such as cognitive decline, polypharmacy, and reduced manual dexterity continue to limit optimal adherence in older adults [11,17,29].

Together, these age- and disease-specific differences highlight a central message emphasized in both guidelines: improving adherence requires tailored strategies that account for developmental stage, symptom patterns, self-management capacity, and treatment complexity. Enhanced and individualized education, combined with systematic patient-centered follow-up, may be necessary to improve therapeutic adherence [30]. A promising solution is the implementation of digital tools, such as mobile applications that centralize patient information, provide disease- and medication-specific educational content, offer personalized medication reminders, and enable secure communication between healthcare professionals and patients [31,32]. These practical interventions align with current GINA and GOLD recommendations and may offer feasible, scalable approaches to support adherence across different age groups.

Sex differences were modest, with slightly lower adjusted odds among females, in line with previous literature [11]. GOLD highlights the role of socioeconomic and behavioral factors, such as health literacy, depression, or lower income, in shaping adherence—elements that may contribute to the observed differences [33,34]. In our sensitivity analysis, year-to-year variation suggests caution against interpreting sex as a fixed determinant (see Appendix A Table A1, Table A2, Table A3, Table A4, Table A5, Table A6, Table A7 and Table A8). However, broader gender-related disparities in healthcare access, highlighted by the European Institute for Gender Equality, may partly explain these trends [35].

Beyond gender-related differences, broader social and economic determinants also play an important role in shaping adherence. Evidence shows higher risks of poor adherence among unemployed, low-income, immigrant patients and those living alone, as well as higher risks of non-use in these same groups [36]. Lower education and low income were likewise associated with increased exacerbations and hospital admissions [36]. Moreover, a recent study reported that triple therapies are more frequently prescribed to patients with lower income and lower educational levels, which may partly explain the higher exacerbation rates observed among socioeconomically disadvantaged groups [37]. Together, these findings indicate that lower socioeconomic status not only undermines therapeutic adherence but also contributes to higher healthcare costs through increased use of more expensive treatments for asthma and COPD and more frequent hospital admissions.

About clinical determinants, a striking variation occurred across pharmacological classes. Adherence to ICS and LABA + ICS—despite their central role in asthma maintenance per GINA recommendations—was alarmingly low (8.1% and 23.5%). This directly mirrors GINA’s observation that ICS have some of the lowest real-world adherence across all asthma medications and that multiple daily doses are a key barrier [38]. GINA explicitly recommends simplifying regimens whenever possible, including using once-daily ICS or LABA + ICS formulations to improve adherence. These findings also mirror earlier Belgian NIHDI data [16], underscoring a persistent gap between guideline-recommended therapy and real-world use.

Adherence to dual bronchodilation (LABA + LAMA) was substantially higher, consistent with international real-world studies [10] and with GOLD’s recognition that simpler bronchodilator regimens tend to achieve better persistence. Triple therapy (ICS + LAMA + LABA) showed the highest adherence (66.4%), with adjusted odds more than ninefold higher than ICS alone. These large effect sizes may partially reflect confounding by disease severity or treatment indication, as patients receiving dual or triple therapy often have more severe or uncontrolled disease, which may increase treatment engagement independently of the regimen itself. This interpretation is consistent with evidence showing improved adherence following exacerbations [39]. This strong association corroborates evidence that single-inhaler triple therapy improves adherence and persistence compared with multiple-inhaler regimens [40,41,42]. GOLD 2025 specifically emphasizes simplifying treatment regimens to reduce inhaler errors, regimen burden, and non-adherence.

Montelukast, an oral therapy, also showed high adherence, reinforcing the broader concept recognized across guidelines that simpler administration routes facilitate more consistent medication use. Although theophylline is no longer recommended by GINA, its relatively high adherence in this cohort illustrates that the simplicity of oral administration remains a powerful behavioral driver across classes and clinical profiles. This does not imply that theophylline should be reconsidered clinically but rather highlights that treatment simplification—whether inhaled or oral—is a facilitator of adherence and should be prioritized whenever safe and clinically appropriate.

A retrospective analysis conducted between 2013 and 2016 using the NIHDI database reported similarly low adherence to ICS and LABA–ICS, with rates of 7.98% and 17.56%, respectively [16]. Compared with these findings, which reported adherence rates of 50.01% for LABA and 62.83% for LAMA, our results from the 2020–2023 period demonstrate similar adherence patterns for these classes (LABA: 53.6%; LAMA: 61.7%) [16]. Furthermore, 50.2% of patients in our cohort were adherent to LABA–LAMA combinations, markedly higher than the 28.6% adherence to tiotropium/olodaterol reported by Moretz et al. between 2015 and 2016 [19]. These consistencies across time periods and healthcare systems reinforce the robustness of our estimates and suggest that the observed pharmacological differences reflect enduring, real-world adherence patterns.

Several factors likely contribute to the pharmacological differences observed, including inhaler complexity, inhalation technique errors, and patient satisfaction with devices. A study conducted between 2020 and 2021 reported a non-adherence rate of 86% among patients using inhaled therapies [43]. Multiple studies have shown that poor inhaler technique and dissatisfaction negatively affect adherence, whereas structured training and digital health tools can significantly improve it [18,44,45]. A promising solution involves the integration of smart inhaler technologies into routine clinical practice. These devices incorporate connected sensors capable of monitoring inhalation technique and tracking dose usage. When linked to mobile health applications, they can provide personalized feedback to patients, thereby supporting improved self-management of chronic respiratory diseases [18].

The most commonly described barriers to therapeutic adherence in asthma include doubts and beliefs regarding the necessity of daily controller medication, with many patients reporting a tendency to take their treatment only when symptoms are present rather than as prescribed [28]. In COPD, low health literacy has been identified as a major obstacle to effective self-management and therapeutic adherence [17]. Patients with COPD commonly report feeling isolated in managing their disease, lacking sufficient information about their condition and its treatments, and experiencing limited motivation to continue long-term inhaled therapies [31]. These behavioral and perceptual barriers highlight that improving adherence requires not only pharmacological optimization but also targeted educational and supportive interventions that address patient beliefs, understanding, and engagement.

Overall, the findings closely align with international evidence showing persistently low adherence to inhaled corticosteroids, despite their central role in asthma management under GINA, and mirror GOLD’s emphasis on treatment simplification as a key strategy to improve long-term persistence. By quantifying these patterns at a national scale and identifying high-risk groups—particularly children, adolescents, and users of ICS or ICS–LABA—this study provides a population-level evidence base to inform health system planning and resource allocation strategies.

Both GINA and GOLD note that self-reported adherence systematically overestimates medication use and recommend objective measures such as dispensing records, dose counters, and electronic monitoring. By quantifying adherence using a standardized, dispensing-based metric (CMA), this study follows guideline-endorsed best practices and avoids the biases inherent to self-report. These findings support the implementation of guideline-concordant interventions, including regimen simplification, structured education, personalized follow-up, enhanced inhaler technique support, and the use of digital tools. Ultimately, integrating these evidence-based strategies into clinical practice may help reduce non-adherence and improve long-term asthma and COPD outcomes.

### Strengths and Limitations

This study benefits from its nationwide scope and complete coverage of reimbursed asthma and COPD medication dispensations through the NIHDI database, ensuring excellent representativeness. The large sample size provides strong statistical power and supports detailed subgroup analysis. The use of a standardized and reproducible adherence metric (CMA3), together with multiyear sensitivity analyses, enhances robustness and methodological transparency. Importantly, the analytical strategy—based on mixed-effects modeling—adds an additional layer of rigor, ensuring that the identified determinants of adherence reflect consistent and statistically robust associations.

However, certain limitations must be considered. Dispensing data do not confirm actual medication intake, meaning measured adherence reflects availability rather than true consumption. This may introduce misclassification, with some patients appearing adherent despite irregular use or incorrect inhalation technique. One limitation is the absence of diagnostic information, which prevents distinguishing between asthma and COPD and makes it impossible to ascertain whether the dispensed medications were truly used for the intended respiratory condition. Although these two conditions share some pharmacological treatments, they have distinct pathophysiological and behavioral profiles that may influence adherence. Because diagnostic coding was unavailable, the selection of patients relied on dispensing patterns; requiring ≥3 dispensations aimed to increase the likelihood of capturing chronic respiratory therapy in line with international asthma and COPD guidelines but may have introduced selection bias by excluding patients with fewer refills, including early non-adherent individuals. At the same time, this criterion also helps exclude individuals who received only a single or sporadic prescription across the 4-year period, who are unlikely to represent true chronic disease management. It was also not possible to distinguish maintenance use from reliever use for some LABA + ICS combinations, particularly formoterol–ICS regimens recommended as as-needed therapy for mild asthma in GINA guidelines since 2019, which may contribute to exposure to misclassification. Finally, reliance on WHO-Defined Daily Doses (DDD) may underestimate exposure in children due to differences between pediatric and adult dosing standards. In the absence of pediatric-specific DDDs, adherence estimates in children should thus be interpreted with caution.

## 5. Conclusions and Perspectives

Future research should build on these nationwide findings to contribute to global efforts aimed at improving adherence to asthma and COPD therapies. Methodological advancements are also required—particularly the development of pediatric-appropriate dosing metrics—to strengthen cross-country comparability of adherence measurements. The integration of clinical data (diagnosis, severity, exacerbations) would enable disease-specific analyses consistent with GOLD and GINA evaluation frameworks and support international benchmarking. Furthermore, because simplified regimens such as single-inhaler triple therapy and oral treatments show the highest adherence, future interventional studies should assess whether regimen simplification or digital adherence technologies can improve continuity of care in line with emerging global trends. Finally, the promising but underused pharmacist-led consultations—such as “New Medicines Service” (NMS), which has been available for asthma since 2013 and was extended to COPD in 2023 in Belgium [46]—warrant formal evaluation using multicentered or multinational implementation science approaches to determine their capacity to improve adherence at scale across different health systems.

Building on these perspectives, our findings highlight important implications for healthcare systems, particularly the need to implement targeted adherence interventions for high-risk groups. Digital tools—such as mobile applications linked to smart inhalers—could support routine adherence monitoring [47] and should be deployed at the health system level. Earlier work in COPD care has shown that both patients and healthcare professionals view such technological platforms positively to support daily disease management and enhance interprofessional collaboration [31]. However, their effectiveness depends on adapting these tools to patient needs, especially for children and adolescents, who display the lowest adherence and face greater management complexity [26,27]. It is therefore essential to explore whether young patients with asthma would similarly perceive these digital solutions as useful and acceptable. To ensure that such interventions are truly patient-centered and age-appropriate, qualitative studies, including interviews and focus groups, are needed to better capture the specific barriers and expectations of these vulnerable populations.

## Figures and Tables

**Table 1:** Baseline characteristics of the study population.

Category	Category	Total Count	Count	Percentage
(a) Sex	Female	1,384,363	727,205	52.5%
	Male		657,158	47.5%
(b) Age Category Distribution	0–10	1,384,363	276,702	20%
	11–20		76,490	5.5%
	21–40		195,365	14.1%
	41–65		449,658	32.5%
	>65		386,148	27.9%
(c) Administration Route	Inhaled	1,384,363	1,231,306	88.9%
	Oral + Inhaled		112,566	8.1%
	Oral		40,491	2.9%
(d) Number of Pharmacological Classes per Patient	1	1,384,363	1,105,548	79.9%
	2		213,116	15.4%
	≥3		65,699	4.8%
(e) Pharmacological Class Distribution	ICS	10,942,658	2,095,912	19.2%
	LABA		270,836	2.5%
	LAMA		550,064	5.0%
	LABA + LAMA		482,641	4.4%
	ICS + LAMA + LABA		691,330	6.3%
	LABA + ICS		5,328,156	48.7%
	Montelukast		1,290,184	11.8%
	Theophylline		233,535	2.1%

a–d: Distributions calculated at the patient level (each patient counted once); e: Distribution calculated at the dispensation level (all recorded dispensations during the study period); ICS: Inhaled Corticosteroids; LABA: Long-Acting Beta-Agonist; LAMA: Long-Acting Muscarinic Antagonist.

**Table 2:** Distribution of adherent and non-adherent patients by demographic and clinical characteristics.

Variable	Category	Total Exposures	% Adherent	% Non-Adherent
All Patients		1,103,386	30.5	69.5
Age Category	0–10	179,569	4.0	96.0
	11–20	46,005	15.7	84.3
	21–40	120,562	21.0	79.0
	41–65	368,832	34.4	65.6
	>65	388,418	43.7	56.3
Sex	Female	568,532	29.9	70.1
	Male	534,854	31.1	68.9
Administration Route	Inhaled	984,755	27.1	72.9
	Oral	118,631	58.9	41.1
Pharmacological Class	ICS	247,502	8.1	91.9
	LABA	23,988	53.6	46.4
	LAMA	50,934	61.7	38.3
	LABA + LAMA	54,045	50.2	49.8
	ICS + LAMA + LABA	75,654	66.4	33.6
	LABA + ICS	532,632	23.5	76.5
	Montelukast	108,250	59.2	40.8
	Theophylline	10,381	55.4	44.6

Total exposures correspond to patient–class combinations rather than unique patients. A single patient can appear in multiple rows if they were dispensed medications from more than one pharmacological class during the OW. Counts therefore represent exposures, not individuals. ICS: Inhaled Corticosteroids; LABA: Long-Acting Beta-Agonist; LAMA: Long-Acting Muscarinic Antagonist.

## Data Availability

The pseudonymized individual-level dataset cannot be shared due to GDPR and contractual restrictions. Only derived, fully anonymized aggregated outputs that support the conclusions of this article can be provided upon reasonable request.

## Abbreviations

The following abbreviations are used in this manuscript:

ANOVA	Analysis of Variance
CI	Confidence Interval
CMA	Continuous Multiple-Interval Measure of Medication Availability
CMA3	Continuous Multiple-Interval Measure of Medication Availability
COPD	Chronic Obstructive Pulmonary Disease
CSI–INAMI	Comité de Sécurité de l’Information–Institut National d’Assurance Maladie-Invalidité
DDD	Defined Daily Dose
DPIA	Data Protection Impact Assessment
FUW	Follow-Up Window
GINA	Global Initiative for Asthma
GOLD	Global Initiative for Chronic Obstructive Lung Disease
ICS	Inhaled Corticosteroids
INAMI	Institut National d’Assurance Maladie-Invalidité (Belgium)
LABA	Long-Acting Beta-Agonist
LAMA	Long-Acting Muscarinic Antagonist
NMS	New Medicines Service
NIHDI	National Institute for Health and Disability Insurance (Belgium)
OR	Odds Ratio
OW	Observation Window
WHO	World Health Organization

## Appendix A. Sensitivity Analysis: Year-by-Year Therapeutic Adherence Among Patients with Asthma and COPD (2020–2023)

**Table A1:** Distribution of adherent and non-adherent patients by demographic and clinical characteristics by year (2020).

2020	Variable	Category	Total Exposures	% Adherent	% Non-Adherent
	All Patients		355,618	61.8	38.2
	Age Category	0–10	18,108	15.6	84.4
		11–20	9787	44.2	55.8
		21–40	27,959	56.3	43.7
		41–65	123,353	64.8	35.2
		>65	176,411	66.4	33.6
	Sex	Female	182,049	61.7	38.3
		Male	173,569	62.0	38.0
	Administration Route	Inhaled	289,465	58.3	41.7
		Oral	66,153	77.4	22.6
	Pharmacological Class	ICS	35,665	30.0	70.0
		LABA	14,834	71.2	28.8
		LAMA	29,450	80.3	19.7
		LABA + LAMA	21,174	67.4	32.6
		ICS + LAMA + LABA	14,121	86.5	13.5
		LABA + ICS	174,221	55.8	44.2
		Montelukast	58,314	78.6	21.4
		Theophylline	7839	67.9	32.1

**Table A2:** Adjusted odds ratios and 95% confidence intervals from the multivariate mixed-effects model (2020).

Year	Variable	Adjusted OR	95% CI
**2020**	Age Category: Children (0–10 years)	0.2	[0.17–0.19]
	Age Category: Adolescents (11–20 years)	0.5	[0.49–0.55]
	Age Category: Adults (41–65 years)	1.3	[1.30–1.38]
	Age Category: Older adults (>65 years)	1.4	[1.40–1.48]
	Sex: Female	0.9	[0.91–0.94]
	Pharmacological Class: Montelukast	7.1	[6.89–7.35]
	Pharmacological Class: ICS + LAMA + LABA	9.1	[8.66–9.66]
	Pharmacological Class: LABA	3.6	[3.48–3.80]
	Pharmacological Class: LABA + ICS	2.0	[1.97–2.08]
	Pharmacological Class: LABA + LAMA	2.9	[2.82–3.05]
	Pharmacological Class: LAMA	5.8	[5.55–5.99]
	Pharmacological Class: Theophylline	3.0	[2.85–3.18]

**Table A3:** Distribution of adherent and non-adherent patients by demographic and clinical characteristics by year (2021).

2021	Variable	Category	Total Exposures	% Adherent	% Non-Adherent
	All Patients		343,179	60.7	39.3
	Age Category	0–10	30,409	13.1	86.9
		11–20	8204	44.7	55.3
		21–40	26,075	57.8	42.2
		41–65	113,147	65.8	34.2
		>65	165,344	67.3	32.7
	Sex	Female	175,008	61.0	39.0
		Male	168,171	60.4	39.6
	Administration Route	Inhaled	286,839	57.5	42.5
		Oral	56,340	77.0	23.0
	Pharmacological Class	ICS	42,204	22.6	77.4
		LABA	11,645	70.5	29.5
		LAMA	24,064	79.8	20.2
		LABA + LAMA	21,936	64.9	35.1
		ICS + LAMA + LABA	25,528	84.1	15.9
		LABA + ICS	161,462	57.2	42.8
		Montelukast	49,223	78.4	21.6
		Theophylline	7117	66.8	33.2

**Table A4:** Adjusted odds ratios and 95% confidence intervals from the multivariate mixed-effects model (2021).

Year	Variable	Adjusted OR	95% CI
**2021**	Age Category: Children (0–10 years)	0.2	[0.19–0.20]
	Age Category: Adolescents (11–20 years)	0.5	[0.52–0.58]
	Age Category: Adults (41–65 years)	1.3	[1.25–1.32]
	Age Category: Older adults (>65 years)	1.4	[1.32–1.39]
	Sex: Female	0.9	[0.91–0.94]
	Pharmacological Class: Montelukast	6.6	[6.40–6.86]
	Pharmacological Class: ICS + LAMA + LABA	7.6	[7.30–7.97]
	Pharmacological Class: LABA	3.5	[3.37–3.72]
	Pharmacological Class: LABA + ICS	2.2	[2.10–2.23]
	Pharmacological Class: LABA + LAMA	2.7	[2.55–2.76]
	Pharmacological Class: LAMA	5.7	[5.43–5.90]
	Pharmacological Class: Theophylline	2.9	[2.73–3.07]

**Table A5:** Distribution of adherent and non-adherent patients by demographic and clinical characteristics by year (2022).

2022	Variable	Category	Total Exposures	% Adherent	% Non-Adherent
	All Patients		347,580	59.4	40.6
	Age Category	0–10	37,849	13.1	86.9
		11–20	8474	42.0	58.0
		21–40	26,326	56.6	43.4
		41–65	111,048	65.3	34.7
		>65	163,883	67.4	32.6
	Sex	Female	178,384	60.0	40.0
		Male	169,196	58.8	41.2
	Administration Route	Inhaled	296,800	56.2	43.8
		Oral	50,780	78.1	21.9
	Pharmacological Class	ICS	51,105	21.7	78.3
		LABA	9307	70.0	30.0
		LAMA	19,573	79.6	20.4
		LABA + LAMA	22,346	64.3	35.7
		ICS + LAMA + LABA	35,279	83.5	16.5
		LABA + ICS	159,190	56.4	43.6
		Montelukast	44,522	79.8	20.2
		Theophylline	6258	66.0	34.0

**Table A6:** Adjusted odds ratios and 95% confidence intervals from the multivariate mixed-effects model (2022).

Year	Variable	Adjusted OR	95% CI
**2022**	Age Category: Children (0–10 years)	0.2	[0.21–0.23]
	Age Category: Adolescents (11–20 years)	0.6	[0.53–0.58]
	Age Category: Adults (41–65 years)	1.3	[1.26–1.34]
	Age Category: Older adults (>65 years)	1.4	[1.37–1.45]
	Sex: Female	0.9	[0.92–0.95]
	Pharmacological Class: Montelukast	7.4	[7.12–7.63]
	Pharmacological Class: ICS + LAMA + LABA	7.6	[7.34–7.93]
	Pharmacological Class: LABA	3.6	[3.41–3.79]
	Pharmacological Class: LABA + ICS	2.2	[2.17–2.30]
	Pharmacological Class: LABA + LAMA	2.7	[2.59–2.79]
	Pharmacological Class: LAMA	5.8	[5.56–6.06]
	Pharmacological Class: Theophylline	2.9	[2.74–3.08]

**Table A7:** Distribution of adherent and non-adherent patients by demographic and clinical characteristics by year (2023).

2023	Variable	Category	Total Exposures	% Adherent	% Non-Adherent
	All Patients		342,363	60.7	39.3
	Age Category	0–10	33,172	15.2	84.8
		11–20	8443	42.8	57.2
		21–40	26,105	56.9	43.1
		41–65	109,079	65.8	34.2
		>65	165,564	67.9	32.1
	Sex	Female	176,969	61.0	39.0
		Male	165,394	60.3	39.7
	Administration Route	Inhaled	294,390	57.6	42.4
		Oral	47,973	79.4	20.6
	Pharmacological Class	ICS	45,188	23.1	76.9
		LABA	7546	71.2	28.8
		LAMA	16,273	80.0	20.0
		LABA + LAMA	24,064	64.5	35.5
		ICS + LAMA + LABA	44,457	83.2	16.8
		LABA + ICS	156,862	56.3	43.7
		Montelukast	42,527	81.1	18.9
		Theophylline	5446	66.1	33.9

**Table A8:** Adjusted odds ratios and 95% confidence intervals from the multivariate mixed-effects model (2023).

Year	Variable	Adjusted OR	95% CI
**2023**	Age Category: Children (0–10 years)	0.3	[0.24–0.27]
	Age Category: Adolescents (11–20 years)	0.6	[0.55–0.62]
	Age Category: Adults (41–65 years)	1.3	[1.25–1.33]
	Age Category: Older adults (>65 years)	1.4	[1.34–1.42]
	Sex: Female	0.9	[0.92–0.95]
	Pharmacological Class: Montelukast	8.0	[7.76–8.33]
	Pharmacological Class: ICS + LAMA + LABA	7.7	[7.46–8.02]
	Pharmacological Class: LABA	4.0	[3.75–4.21]
	Pharmacological Class: LABA + ICS	2.3	[2.25–2.38]
	Pharmacological Class: LABA + LAMA	2.8	[2.71–2.92]
	Pharmacological Class: LAMA	6.2	[5.89–6.47]
	Pharmacological Class: Theophylline	3.0	[2.84–3.21]

Table A1, Table A3, Table A5 and Table A7: Total Exposures represent patient-class combinations within the CMA3 subset. A single patient may appear multiple times if they were dispensed medications from more than one pharmacological class during the Observation Window. ICS: Inhaled Corticosteroids; LABA: Long-Acting Beta-Agonist; LAMA: Long-Acting Muscarinic Antagonist; N: Number of Patients.

Table A2, Table A4, Table A6 and Table A8: OR = Odds Ratio (adjusted for Age Category, Sex, and Pharmacological Class); Reference categories: Age Category: 21–40 years; Sex: Male; Pharmacological Class: ICS. All associations presented in the table are statistically significant (*p* < 0.001). OR: Odds Ratios; CI: Confidence Interval; ICS: Inhaled Corticosteroids; LABA: Long-Acting Beta-Agonist; LAMA: Long-Acting Muscarinic Antagonist.

## Appendix B. Adjusted Odds Ratios and 95% Confidence Intervals from the Multivariate Mixed-Effects Model

**Table A9:** Adjusted odds ratios and 95% confidence intervals from the multivariate mixed-effects model.

Variable	*p*-Value	Adjusted OR	95% CI
Age Category: Children (0–10 years)	<0.001	0.25	[0.24–0.26]
Age Category: Adolescents (11–20 years)	<0.001	0.59	[0.58–0.61]
Age Category: Adults (41–65 years)	<0.001	1.70	[1.67–1.73]
Age Category: Older adults (>65 years)	<0.001	2.42	[2.38–2.46]
Sex: Female	<0.001	0.89	[0.89–0.90]
Pharmacological Class: Montelukast	<0.001	11.02	[10.79–11.25]
Pharmacological Class: ICS + LAMA + LABA	<0.001	9.09	[8.89–9.30]
Pharmacological Class: LABA	<0.001	5.51	[5.34–5.68]
Pharmacological Class: LABA + ICS	<0.001	1.80	[1.77–1.84]
Pharmacological Class: LABA + LAMA	<0.001	4.49	[4.38–4.60]
Pharmacological Class: LAMA	<0.001	7.08	[6.90–7.25]
Pharmacological Class: Theophylline	<0.001	5.36	[5.13–5.60]

Reference categories: Age Category: 21–40 years; Sex: Male; Pharmacological Class: ICS. All associations presented in the table are statistically significant (*p* < 0.001). OR: Odds Ratios; CI: Confidence Interval; ICS: Inhaled Corticosteroids; LABA: Long-Acting Beta-Agonist; LAMA: Long-Acting Muscarinic Antagonist.
